# The Treatment of Bronchiectasis

**Published:** 1902-04-26

**Authors:** 


### THE TREATMENT OF BRONCHIECTASIS.

After a very full and careful discussion of the clinical and pathological aspects of bronchiectasis Dr. Theodore Dyke Acland[1] comes to the subject of treatment. The various methods employed he places in the order of their efficiency as follow:—

1. Inhalation of volatalised antiseptics: (*a*) creosote vapour-baths; (*b*) inhalations of creosote, oil of peppermint, eucalyptus, etc.
2. Subcutaneous and intravenous injections of antiseptic fluids.
3. Internal medication.
4. Surgical treatment (incision and drainage).

Sometimes one and sometimes another of these methods seems to succeed in a particular case and fails hopelessly to give relief in another. The most successful means of relieving the fœtor, and occasionally, but by no means always, of lessening the amount of expectoration, is the inhalation of crude creosote vapour in the form of a creosote vapour bath. It is a disagreeable process causing much cough and a considerable amount of distress. By the cough, however, "not only are the tubes cleared of the offensive secretions, but the deep inspirations which follow serve to draw the creosote vapour into the smaller bronchioles, bringing it into immediate contact with the decomposing pus in the dilated tubes." To this is doubtless due much of the efficacy of the method.

Ordinary commercial creosote is used. It is to be vapourised in an "evaporating dish of the capacity of half a pint," over a spirit lamp, in a small room arranged for the purpose, and it must be remembered that "the volatalisation of a large amount of crude creosote makes everything in a mess" and that it may be necessary to protect the eyes and face with a mask, though some patients prefer to do this with a handkerchief only. The ordinary inhalations used with a perforated zinc respirator are merely make-shifts and are doomed to failure in any case really requiring energetic treatment.

Of intra-laryngeal medication—the injection into the trachea of considerable quantities of medicated fluids—Dr. Acland's experience has not been favourable. Nor has he much good to say either of subcutaneous injections of guiacol, creosote, etc., or of intravenous injection of formalin.

As to the surgical treatment of bronchiectasis, Dr. Acland says that in any case in which there are signs of a large cavity there is naturally a great temptation to treat it by incision and drainage. But "there is nothing more melancholy than the ultimate results of operations for the drainage of bronchiectatic cavities." It must be remembered, however, that in some cases the diagnosis is not absolutely clear, and that the less certain one is that the case is one of pure and simple bronchietasis the more hopeful may be the prospects of an operation. "The more probability there is of the symptoms being due to (*a*) pulmonary abscess; (*b*) empyæma, subphrenic abscess; or (*c*) hepatic abscess, or hydatid, the more justification there is for an exploratory operation."

Lastly, we come to a method which, when the cavities are large or numerous and the secretions not too viscid, may give considerable relief—namely, that of emptying the dilated tubes by the inversion of the patient. This must be done with care, and the patient must be turned towards the most affected side, so as to avoid flooding the less damaged lung. It may reasonably be expected that when the power of clearing the tubes by coughing is very small relief may be obtainable in this manner which would be difficult to gain in any other way.

When bronchiectasis is once fairly established in the adult it is incurable, except in very rare instances. In chronic cases, however, the distress can often be greatly relieved, and, if the exciting cause be not progressive, life can be much prolonged.

[1] Practitioner, April.

